# Different Proteome Profiles between Male and Female *Populus cathayana* Exposed to UV-B Radiation

**DOI:** 10.3389/fpls.2017.00320

**Published:** 2017-03-07

**Authors:** Yunxiang Zhang, Lihua Feng, Hao Jiang, Yuanbin Zhang, Sheng Zhang

**Affiliations:** ^1^Key Laboratory of Mountain Surface Processes and Ecological Regulation, Institute of Mountain Hazards and Environment, Chinese Academy of SciencesChengdu, China; ^2^University of Chinese Academy of SciencesBeijing, China; ^3^Institute of Evolution and the Department of Evolutionary and Environmental Biology, University of HaifaHaifa, Israel

**Keywords:** dioecious, plant proteomics, UV-B radiation, sexual difference, poplar

## Abstract

With increasing altitude, solar UV-B radiation is enhanced. Based on the phenomenon of male-biased sex ratio of *Populus cathayana* Rehder in high altitude alpine area, we hypothesized that males have a faster and more sophisticated responsive mechanism to high UV-B radiation than that of females. Our previous studies have shown sexually different responses to high UV-B radiation were existed in *P. cathayana* at the morphological, physiological, and transcriptomic levels. However, the responses at the proteomic level remain unclear. In this study, an isobaric tag for relative and absolute quantification (iTRAQ)-based quantitative proteome analysis was performed in *P. cathayana* females and males. A total of 2,405 proteins were identified, with 331 proteins defined as differentially expressed proteins (DEPs). Among of these, 79 and 138 DEPs were decreased and 47 and 107 DEPs were increased under high solar UV-B radiation in females and males, respectively. A bioinformatics analysis categorized the common responsive proteins in the sexes as related to carbohydrate and energy metabolism, translation/transcription/post-transcriptional modification, photosynthesis, and redox reactions. The responsive proteins that showed differences in sex were mainly those involved in amino acid metabolism, stress response, and translation/transcription/post-transcriptional modification. This study provides proteomic profiles that poplars responding to solar UV-B radiation, and it also provides new insights into differentially sex-related responses to UV-B radiation.

## Introduction

Natural levels of ultraviolet-B (UV-B: 280–320 nm) radiation act as an environmental regulatory factor in plants and control gene expression, growth and development (Jenkins, 2009; Hideg et al., 2013; Czégény et al., 2016). In alpine areas, with increasing altitude, UV-B radiation is enhanced. High doses of UV-B radiation negatively affect cellular/subcellular and macromolecular structures in vegetative plant tissues, including DNA, RNA, and proteins, modulate metabolites and reduce photosynthesis, biomass, and seed production (Jansen et al., 1998; Ries et al., 2000). Thus, plants must sense natural UV-B intensity and quickly respond to high levels of UV-B radiation as an acclimation process (Jenkins, 2009). A number of studies have reported that the responsive and adaptive mechanisms of plants to high altitude with high UV-B radiation differ among species, populations and genotypes (Ren et al., 2006, 2007; Wang et al., 2016). However, few studies have focused on the sexual differences in the responses.

In nature, the sex ratio of *Populus* is consistent with a 1:1 equilibrium in low-altitude habitats (1,600–2,000 m). However, at high altitude (2,000–2,600 m), there is a significant male-biased sex ratio (Wang et al., 2011; Lei et al., 2017). Therefore, we hypothesized that males are more resistant and have a more sophisticated response to high UV-B radiation than that of females, thus leading to a male-biased sex ratio at high altitude. To test this hypothesis, based on previous work, we exposed *Populus cathayana* Rehder to different UV-B radiation intensities and showed differences in the sexual responses to high UV-B radiation (Xu et al., 2010; Feng et al., 2014; Jiang et al., 2015). At the physiological level, males appear to be more resistant to high UV-B radiation. Observations of the organelle ultrastructure, photosynthetic rate and biomass accumulation indicated that these parameters were less affected by increased UV-B intensity in males than in females (Xu et al., 2010; Feng et al., 2014), and UV-B exposure enhanced bud break in male clones (Stromme et al., 2015). A comparative transcriptome analysis indicated that in *P. cathayana* males, sex-related transcriptional reprogramming occurred in certain important metabolic processes, and showed that sex-biased gene regulation under solar UV-B radiation was more responsive in males than in females (Jiang et al., 2015). At the metabolic level, UV-B radiation has been reported to mediate metabolic rearrangement in gray poplars (*P. canescens* syn.; Kaling et al., 2015), and increased UV radiation has been shown to promote the emission of more volatile organic compounds in females of European aspen (*P. tremula* L.) than in male plants (Randriamanana et al., 2015; Maja et al., 2016). However, sexual differences in the response to UV-B radiation at the proteomic level have not been reported. Because of the complicated and sophisticated modification and regulation of gene expression processes in plant cells, UV-B radiation may promote different patterns of change in the pathways at the mRNA, protein and metabolite levels. Therefore, determining how plants respond to high UV-B radiation at the leaf proteomic level will provide new insights into UV-B responsive processes and ultimately increase our understanding of the sex-ratio variation in high-altitude alpine areas. In this study, an iTRAQ-based quantitative proteome analysis was performed. The objectives were to (1) understand how UV-B radiation triggers overall proteomic changes and (2) assess whether males are more responsive than females to high UV-B radiation.

## Materials and methods

### Plant materials and experimental design

The material treatment and experimental design were as described by Jiang et al. (2015). Briefly, male and female cuttings were produced from F_1_ individuals derived from a controlled cross between two *P. cathayana* genotypes with divergent phenotypes. Sixty male and female cuttings were planted in 10 L plastic pots (one plant per pot) filled with 8 kg of homogenized soil and 8 g of slow-release fertilizer (13% N, 10% P, and 14% K) in a greenhouse located in the Wanglang National Nature Reserve (32°98′N and 104°08′E, Supplementary Figure 1). The altitude, mean annual rainfall, annual temperature, and annual summer temperature in the area are 2,600 m, 801 mm, 2.9 and 12.7°C, respectively.

**Figure 1:**
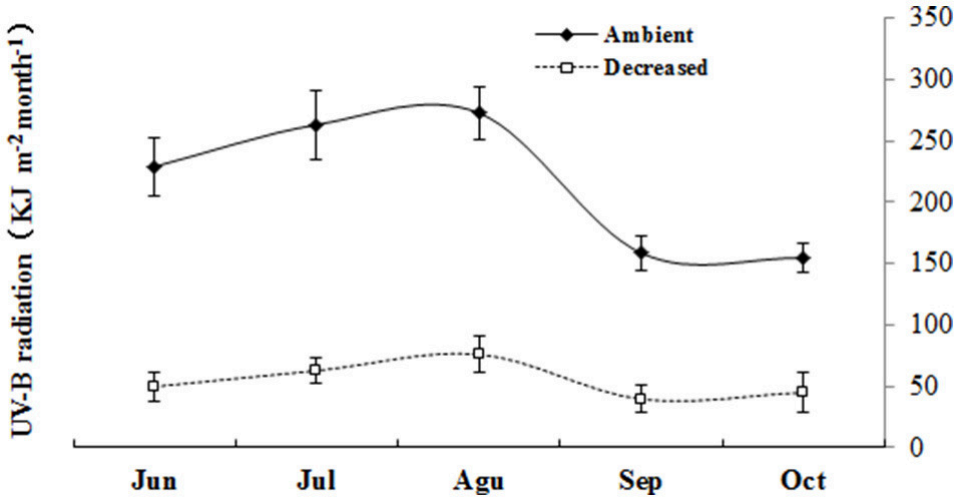
**Monthly UV-B radiation accumulation of the two different UV-B radiation intensities in the experimental site during June to October**.

Two UV-B radiation intensities were used: 2.1 and 8.5 kJ m^−2^ day^−1^ of average daily biologically effective UV-B (UV-BBE) radiation. In the low solar UV-B radiation treatment (2.1 kJ m^−2^ day^−1^), a polyester film (0.13 mm, Shanghai HiTeC Plastics Co. Ltd., Shanghai, China) was used to selectively exclude solar transmission, including UV-B and UV-C radiation. In the high solar UV-B radiation treatment (8.5 kJ m^−2^ day^−1^), 0.13 mm cellulose diacetate film (Qingzhou Yi-Run Agricultural Film Factory, Qingzhou, Shandong, China) was employed, which allowed the transmission of both UV-A and UV-B radiation (wavelength ≥290 nm). The spectral irradiance was weighted using the generalized plant response function normalized at 300 nm to obtain the UV-BBE radiation (Caldwell, 1971). The polyester and cellulose diacetate films were both replaced weekly. The spectral irradiance of the solar radiation at the plant level was determined using a USB2000 Fiber Optic Spectrometer (Ocean Optics, Inc., Dunedin, New Zealand) with a CC-3-UV Cosine Corrector. Prior to the measurements, the spectrometer was calibrated using a DH2000-CAL Radiometric Calibrated Deuterium Tungsten Source (210–1,050 nm, OceanOptics, Inc., Minneola, Florida, US). Figure 1 shows the high and low solar UV-B radiation intensities from June to October 2012.

The experimental layout was completely randomized with two factors: sex and UV-B radiation. Therefore, there were four treatments: (1) males exposed to low solar UV-B radiation (MC), (2) females exposed to low solar UV-B radiation (FC), (3) males exposed to high solar UV-B radiation (MU), and (4) females exposed to high solar UV-B radiation (FU). The duration of the experiments was 100 days (from 1 June to 10 August).

### Gas exchange and chlorophyll fluorescence measurements

Five cuttings of the 4th fully expanded leaves from each sex per treatment were randomly selected for the gas exchange and chlorophyll fluorescence measurements. The net photosynthesis rate (*P*_*n*_), stomatal conductance (*g*_*s*_), intercellular CO_2_concentration (*C*_*i*_), and transpiration rate (*E*) were measured using a LI-COR 6400 portable photosynthesis measuring system (LI-COR, Lincoln, NE, USA) between the hours of 08:30 and 11:30. Prior to conducting the measurements, the samples were illuminated with saturated PPFD provided by the LED light source of the equipment for 10 min to achieve full photosynthetic induction. The saturated photosynthetic photon flux density (PPFD) was determined by preliminary experiments, and a carbon dioxide gas cylinder (LI-COR, Lincoln, NE, USA) was used to provide constant and stable CO_2_ during the measurements. A standard LI-COR leaf chamber (2 × 3 cm) was used, and the optimal parameters were as follows: leaf temperature of 25°C, leaf-air vapor pressure deficit of 1.5 ± 0.5 kPa, CO_2_ concentration of 400 ± 5 μmol mol^−1^, and photosynthetic photon flux density of 1,400 mmol m^−2^ s^−1^.

Chlorophyll fluorescence was measured using a PAM chlorophyll fluorometer (PAM 2100, Walz, Effeltrich, Germany). The leaf samples were placed in the dark for 30 min using an aluminum foil cover, followed by measurement of the minimal fluorescence (*F*_*o*_) and the maximal fluorescence (*F*_*m*_). Then, the leaves were illuminated with actinic light at an intensity of 250 μmol m^−2^ s^−1^. The actinic light was removed and the minimal fluorescence ($F_{o}^{\prime }$) and the maximal fluorescence ($F_{m}^{\prime }$) were measured by illuminating the leaves with far-red light for 3 s. Finally, a saturating white light with a pulse of 8,000 μmol m^−2^ s^−1^ was applied for 0.8 s to measure the *F*_*m*_ and $F_{m}^{\prime }$ values. The measurements were conducted from 07:30 to 09:00. The chlorophyll fluorescence kinetics parameters (Fv/Fm, maximum efficiency of PSII; *Yield*, the effective quantum yield of PSII; *qP*, photochemical quenching coefficient; *qN*, non-photochemical quenching coefficient) were measured and calculated as described by Vankooten and Snel (1990).

### Leaf transmission electron microscopy

For transmission electron microscopy analysis, small leaf sections (2 mm in length; avoiding the midrib) were selected from the middle position of the leaves. Five biological replicates were used. Leaf sections were fixed in 2.5% (v/v) glutaralpentanedial in 0.2 M phosphate buffered saline (pH 7.0) for 3 h at 22°C and post-fixed in 2% osmium tetroxide (OsO_4_) for 2 h. Then, the leaves were sequentially dehydrated in 30, 50, 70, and 90% acetone, respectively, and embedded in Epon 812 for ~2 h. Ultra-thin sections (80 nm) were sliced, stained with uranyl acetate and lead citrate, and mounted on copper grids for viewing in a H-600IV TEM (Hitachi, Tokyo, Japan) at an accelerating voltage of 60.0 kV.

### Hydrolyzed amino acid compound measurements

Powdered dried leaves (0.1 g) were transferred into a 20-ml hydrolysis tube with 10 ml of 6 mol l^−1^ hydrochloric acid. The hydrolysis tube was sealed under vacuum and transferred to a constant-temperature drier at 110 ± 2°C for 22 h. After the tubes were removed from the dryer and cooled, the hydrolysis liquid was filtered, transferred into a 50-ml volumetric flask, and diluted with deionized water to scale. Then, 1 ml of the diluted hydrolysis liquid was withdrawn and dried in a vacuum drier at 40–50°C. The residue was dissolved in 1 ml of sodium citrate-hydrochloric acid buffer solution (pH 2.2) for analysis. The concentration and composition of amino acids were determined by an L-8800 automatic amino acid analyzer (Hitachi, Tokyo, Japan).

### Protein extraction, digestion, and iTRAQ labeling

Total proteins were extracted from three biological replicates (fresh leaves from tree cuttings) in each treatment using acetone methods as previously described (Zhang S. et al., 2016). The leaves were ground to a fine powder and suspended in 0.5 M triethylammonium bicarbonate (TEAB) buffer with 1 mM phenylmethyl sulfonyl fluoride and 0.1% SDS (w/v), and the samples were then sonicated for 5 min and centrifuged at 25,000 g for 20 min. The supernatant was transferred to another tube, 0.5 M TEAB buffer was added to the pellet to repeat the protein extraction, and the sample was centrifuged at 25,000 g for 20 min. The proteins in the combined supernatant were reduced (10 mM DTT, 56°C for 60 min), alkylated (55 mM iodoacetamide, dark room temperature for 45 min), precipitated by pre-cooled acetone at −20°C for 2 h, and then centrifuged at 25,000 g for 20 min. The pellet was washed twice with acetone, and the final pellet was dissolved in 0.5 M TEAB buffer with 0.1% SDS, sonicated for 15 min, and centrifuged at 25,000 g for 20 min. The supernatant was used for liquid digestion, and the protein concentration was determined using the Bradford assay.

The processed protein (100 μg) was removed from each sample solution and digested with Trypsin Gold (Promega, Madison, WI, USA) at a protein: trypsin ratio of 20:1 at 37°C for 12 h. After trypsin digestion, the peptides were dried by vacuum centrifugation, followed by reconstitution in 0.5 M TEAB and processing according to the manufacturer's protocol for 8-plex iTRAQ (Applied Biosystems). In this work, three biological replicates from each treatment and sex were analyzed. Thus, 12 samples were divided into two sets for iTRAQ. Males exposed to high (MU) and low (MC) solar UV-B radiation were in set 1, and females exposed to high (FU) and low (FC) solar UV-B radiation were in set 2. The MC1 samples were labeled in both sets as the control group. The detailed labeling conditions are listed in Table 1.

**Table 1:** **The labeling strategy used for iTRAQ**.

**Labeling**	**Set 1**		**Set 2**	
	**Treatment**	**Protein content (mg g^−1^ Fw)**	**Treatment**	**Protein content (mg g^−1^ Fw)**
113	MC1	25.33	MC1	25.33
114	MC2	24.87	FC1	24.47
115	MC3	24.01	FC2	26.15
116	MU1	23.05	FC3	25.50
117	MU2	25.73	FU1	26.18
118	MU3	26.27	FU2	24.75
119			FU3	25.44

*MC, males under low UV-B radiation; FC, females under low UV-B radiation; MU, males under high UV-B radiation; FU, males under high UV-B radiation; Fw, fresh weight*.

### Separation of peptides by strong cation exchange (SCX) and ESI mass spectrometric analysis

The labeled samples were fractionated using a LC-20AB high-performance liquid chromatography (HPLC) system (Shimadzu, Kyoto, Japan) and a 4.6 × 250 mm Ultremex strong cation exchange (SCX) column (Phenomenex, Torrance, CA, USA). After reconstitution of the labeled peptide mixtures with 4 ml of buffer A [10 mM NaH_2_PO_4_ in 25% acetonitrile (CAN, v/v), pH 2.6], SCX separation was performed at a flow rate of 1 ml min^−1^ using elution buffer A for 10 min, followed by a linear gradient of 5–60% buffer B (25 mM NaH_2_PO_4_, 1 M KCl in 25% ACN, pH 2.7) for 20 min and 100% buffer B for 2 min. The column was equilibrated with buffer A for 10 min prior to the next injection. The eluted fractions were monitored by measuring the absorbance at 214 nm, desalted with a Strata X C18 column (Phenomenex, Torrance, CA, USA), and vacuum dried.

Each fraction was resuspended in buffer A [5% ACN, 0.1% FA (v/v)] and centrifuged at 20,000 g for 10 min. The final concentration of peptide was ~0.5 μg μl^−1^. On average, 10 μl supernatant was loaded on a LC-20 AD nanoHPLC (Shimadzu, Kyoto, Japan) by an auto sampler onto a 2 cm C18 trap column. The peptides were eluted onto a 10-cm analytical C18 column (inner diameter 75 μm) packed in-house. The samples were loaded at 8 μl min^−1^ for 4 min. Then, a 35-min gradient was run at 300 nl min^−1^ from 2 to 35% B (95% ACN, 0.1% FA), followed by a 5-min linear gradient to 60% (v/v), a 2-min linear gradient to 80%, maintenance at 80% B for 4 min, and 5% for 1 min.

MS analysis was performed using a Triple TOF 5600 System (AB SCIEX, Concord, ON) fitted with a Nanospray III source (AB SCIEX, Concord, ON) and a pulled quartz tip as the emitter (New Objectives, Woburn, MA). The data were acquired using an ion spray voltage of 2.5 kV, curtain gas of 30 psi, nebulizer gas of 15 psi, and an interface heater temperature of 150°C. The MS was operated with an RP ≥30,000 FWHM for the TOF MS scans. For the IDA, survey scans were acquired in 250 ms, and as many as 30 product ion scans were collected if a threshold of 120 counts per second (counts s^−1^) was exceeded at a 2+ to 5+ charge state. The total cycle time was fixed to 3.3 s. The Q2 transmission window was 100 Da for 100%. Four time bins were summed for each scan at a pulse frequency value of 11 kHz via monitoring of the 40 GHz multichannel TDC detector with a four-anode channel detection ion. A sweeping collision energy setting of 35 ± 5 eV coupled with iTRAQ-adjusted rolling collision energy was applied to all pre-cursor ions for collision-induced dissociation. In this experiment, full MS scans were acquired in a mass range of m/z 350 to 1,500 in a scan time of 250 ms. Fragment ion spectra were acquired in the mass range of m/z 100–2,000 and excluded for further fragmentation over 15 s.

### Protein identification and quantification

The raw data files were converted into MGF files and then searched against a local poplar database (ftp://ftp.jgi-psf.org/pub/compgen/phytozome/v9.0/Ptrichocarpa/annotation/) using the Mascot server (version 2.3.02, Matrix Science, Boston, MA). The data were downloaded from *Populus trichocarpa* V 3.0 (73013 protein-coding transcripts; https://phytozome.jgi.doe.gov/pz/portal.html#!info?alias=Org_Ptrichocarpa). The Mascot search settings were as follows: one missed cleavage site by trypsin was allowed with a fixed modification of carbamidomethyl (C), iTRAQ8plex (N-term) and iTRAQ8plex (K) and variable modifications of Gln- > pyro-Glu (N-term Q), oxidation (M), and iTRAQ8plex (Y). The fragment mass tolerance was ±0.1 Da, and the peptide mass tolerance was ±0.05 Da. The max missed cleavages value was 1. The specified false discovery rate (FDR) was 1% when automatic searching using software of pFind 2.0 (Institute of Computing Technology, Chinese Academy of Sciences, Beijing, China). The detailed information of pFind can be referenced to Wang et al. (2007) and Li et al. (2005). For protein identification, at least 2 unique peptides identified were considered. For proteins that were not identified in the local poplar database, SwissProt database (August, 2016, including 553,474 sequences) was used for re-searching using the same parameter settings. The ratio of treatments to controls over ±1.5 and *P* ≤ 0.05 (*t*-test) were considered to indicate the differentially expressed proteins (DEPs). The proteins identified within a family were grouped in the Mascot protein family summary. The sample labeled 113 (MC1) was used as the reference (the control group) based on the weighted average of the intensity of report ions in each identified peptide. The final ratios of protein were then normalized by the median average protein ratio for the mixes of different labeled samples. This normalization corrects the systematic error. For each sex, only DEPs identified in all biological replicates (including the three controls and three UV-B treatments) were further used in the analysis of sexually differential expression. DEPs that were detected in both sexes were defined as commonly changed DEPs, and those detected in only one gender were defined as sexual DEPs. All of the DEPs were functionally categorized using Blast2go, a web-based bioinformatics tool that groups proteins based on their GO annotations. According to the molecular functions listed on the UniProt and Gene Ontology websites, the DEPs were classified into different functional categories in both sexes.

### Statistical analysis

For the physiological parameters, the effects of UV-B radiation, sex and their interaction were analyzed by an analysis of variance (ANOVA) using a randomized complete-block design in SPSS 16.0 (SPSS, Chicago, IL, USA). Prior to the analysis, the data were checked for normality and homogeneity of variance. *Post-hoc* comparisons were tested using Tukey's test at a significance level of *P* < 0.05. The mean values and standard errors were determined for each variable. For protein identification, at least 2 unique peptides identified were considered. The ratio of treatments to controls over ±1.5 and *P* ≤ 0.05 were considered to indicate the DEPs.

## Results

### Gas exchange and chlorophyll fluorescence parameter changes in both sexes

Overall, compared with the low solar UV-B radiation treatment, the high solar UV-B radiation significantly reduced the *P*_*n*_, *g*_*s*_, *C*_*i*_, *E, qP*, and chlorophyll fluorescence *Yield* values in females but not in males. For all parameters, the interactive effects of sex and UV-B were significant (Table 2). Additionally, the *g*_*s*_, *C*_*i*_, *E, qP*, and *Yield* values showed significant sexual differences under the high UV-B radiation treatment but not under the low UV-B radiation treatment.

**Table 2:** **Gas exchange and chlorophyll fluorescence parameters in female and male ***P. cathayana*** cuttings as affected by solar UV-B radiation**.

**Treatment**	***P*_*n*_(μmol m^−2^ s^−1^)**	***g*_*s*_(mol m^−2^ s^−1^)**	***C*_*i*_(μmol mol^−1^)**	***E* (mmol m^−2^ s^−1^)**	***Yield***	***qP***	***qN***	**Fv/Fm**
MC	19.55 ± 0.42^b^	0.64 ± 0.04^b^	314.40 ± 5.10^b^	6.32 ± 0.05^b^	0.70 ± 0.01^ab^	0.96 ± 0.00^b^	0.42 ± 0.03^a^	0.84 ± 0.00^c^
FC	18.46 ± 0.61^b^	0.53 ± 0.03^b^	302.11 ± 3.13^b^	6.00 ± 0.08^b^	0.69 ± 0.03^ab^	0.92 ± 0.02^b^	0.30 ± 0.07^a^	0.82 ± 0.01^ab^
MU	17.72 ± 0.81^ab^	0.64 ± 0.02^b^	308.36 ± 1.28^b^	6.19 ± 0.13^b^	0.74 ± 0.01^b^	0.96 ± 0.00^b^	0.27 ± 0.05^a^	0.83 ± 0.00^bc^
FU	15.51 ± 0.24^a^	0.34 ± 0.04^a^	283.02 ± 7.08^a^	4.93 ± 0.28^a^	0.62 ± 0.04^a^	0.84 ± 0.04^a^	0.26 ± 0.05^a^	0.81 ± 0.00^a^
F: *sex*	0.000	0.012	0.182	0.002	0.010	0.000	0.230	0.000
F: *uvb*	0.332	0.010	0.016	0.010	0.610	0.046	0.092	0.007
F: *sex* × *uvb*	0.009	0.000	0.001	0.000	0.024	0.028	0.354	0.427

*Each value is the mean ± SE (n = 5). MC, males under low UV-B radiation; MU, males under high UV-B radiation; FC, females under low UV-B radiation; FU, females under high UV-B radiation; F_sex_, sex effect; F _uvb_, UV-B effect; F_sex_×_uvb_, the interactive effect of sex and UV-B. Within a column, values followed by different letters are significantly different at P < 0.05 according to Tukey's test. P_n_, the net photosynthesis rate; g_s_, stomatal conductance; C_i_, intercellular CO_2_ concentration; E, transpiration rate; Yield, the effective quantum yield of PSII; qP, photochemical quenching coefficient; qN, non-photochemical quenching coefficient; Fv/Fm, maximum efficiency of PSII*.

### Ultrastructural morphological changes in both sexes

**Figure 2:**
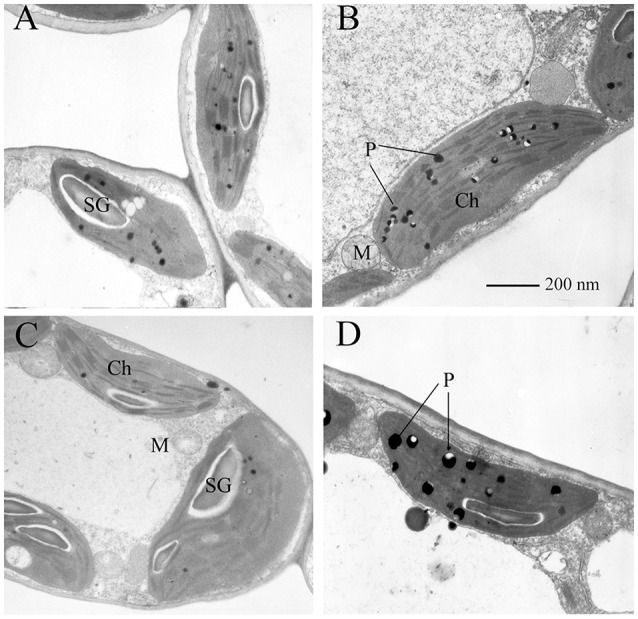
**Transmission electron micrographs of the mesophyll cells of ***P. cathayana*** males and females exposed to UV-B radiation. (A)** males under low UV-B radiation; **(B)** males under high UV-B radiation; **(C)** females under low UV-B radiation; **(D)** females under high UV-B radiation; *Ch*, chloroplast; *M*, mitochondrion; *P*, plastoglobule; *SG*, starch grain.

As shown in Figure 2, under low UV-B radiation, the chloroplasts of both sexes had a typical ellipsoidal shape and typical structure, and 15–30 well-arranged thylakoids (on average 20) were included in each granum (Figures 2A,C). Under high UV-B radiation, the number of starch grains was lower (Figures 2B,D) than that in the individuals under the low UV-B conditions, and additional plastoglobuli were present in the chloroplasts. The accumulation of plastoglobuli is observed as lipid droplets derived from thylakoid degradation, and it was greater in females (Figure 2D) than in males (Figure 2B). However, the high intensity of UV-B radiation did not cause significant changes in the chloroplast granum number, mitochondria, or cellular membrane in the ultra-structures of the mesophyll cells.

### Hydrolyzed amino acid compound changes in both sexes

In this study, a total of 17 types of amino acids were detected in both males and females (Table 3). High UV-B radiation significantly decreased the aspartic acid, leucine, arginine, and proline concentrations, whereas cystine exhibited smaller changes in both sexes. Interestingly, the other amino acids were significantly decreased in males but not in females under high UV-B radiation.

**Table 3:** **The hydrolyzed amino acid concentrations in ***P. cathayana*** female and male leaves as affected by solar UV-B radiation**.

**Amino acids (mg g^−1^ DW)**	**MC**	**MU**	**FC**	**FU**	**F: *sex***	**F: *uvb***	**F: *sex*×*uvb***
Aspartic acid	1.44 ± 0.01^c^	1.16 ± 0.00^*d*^	1.67 ± 0.02^a^	1.63 ± 0.00^b^	0.000	0.000	0.000
Threonine	0.66 ± 0.00^b^	0.52 ± 0.00^c^	0.77 ± 0.00^a^	0.76 ± 0.00^a^	0.000	0.001	0.000
Serine	0.68 ± 0.00^b^	0.55 ± 0.01^c^	0.77 ± 0.01^a^	0.76 ± 0.00^a^	0.000	0.000	0.000
Glutamic acid	1.85 ± 0.00^b^	1.53 ± 0.00^c^	2.16 ± 0.01^a^	2.16 ± 0.00^a^	0.000	0.000	0.000
Glycine	0.81 ± 0.01^b^	0.64 ± 0.00^c^	0.94 ± 0.02^a^	0.92 ± 0.00^a^	0.000	0.000	0.000
Alanine	1.14 ± 0.00^b^	0.90 ± 0.00^c^	1.34 ± 0.00^a^	1.34 ± 0.00^a^	0.000	0.000	0.000
Cystine	0.06 ± 0.00^a^	0.06 ± 0.00^a^	0.06 ± 0.00^a^	0.06 ± 0.00^a^	0.408	0.061	0.119
Valine	0.87 ± 0.01^b^	0.70 ± 0.00^c^	1.03 ± 0.01^a^	1.03 ± 0.00^a^	0.000	0.000	0.000
Methionine	0.14 ± 0.00^b^	0.10 ± 0.01^c^	0.20 ± 0.01^a^	0.20 ± 0.00^a^	0.000	0.000	0.000
Isoleucine	0.67 ± 0.00^b^	0.52 ± 0.00^c^	0.78 ± 0.01^a^	0.79 ± 0.00^a^	0.000	0.001	0.000
Leucine	1.27 ± 0.00^c^	0.97 ± 0.00^*d*^	1.58 ± 0.01^a^	1.55 ± 0.00^b^	0.000	0.000	0.000
Tyrosine	0.35 ± 0.01^b^	0.25 ± 0.00^c^	0.44 ± 0.00^a^	0.44 ± 0.01^a^	0.000	0.000	0.000
Phenylalanine	0.75 ± 0.00^b^	0.58 ± 0.00^c^	0.92 ± 0.00^a^	0.91 ± 0.00^a^	0.000	0.000	0.000
Lysine	0.49 ± 0.01^a^	0.35 ± 0.00^b^	0.49 ± 0.01^a^	0.49 ± 0.00^a^	0.000	0.000	0.000
Histidine	0.37 ± 0.00^b^	0.32 ± 0.01^c^	0.44 ± 0.01^a^	0.43 ± 0.00^a^	0.000	0.000	0.000
Arginine	0.56 ± 0.00^c^	0.43 ± 0.00^d^	0.70 ± 0.01^a^	0.68 ± 0.00^b^	0.000	0.000	0.000
Proline	0.66 ± 0.01^c^	0.56 ± 0.00^d^	0.83 ± 0.01^a^	0.81 ± 0.00^b^	0.000	0.000	0.000

*MC, males under low UV-B radiation; MU, males under high UV-B radiation; FC, females under low UV-B radiation; FU, females under high UV-B radiation; DW, dried weight; F_sex_, sex effect; F_uvb_, UV-B effect; F_sex_×_uvb_, the interactive effect of sex and UV-B; Values followed by different letters in the same row are significantly different at the P < 0.05 level according to Tukey's test. Values are mean ± SE (n = 3)*.

### Protein expression profiles in response to solar UV-B radiation in both sexes

**Figure 3:**
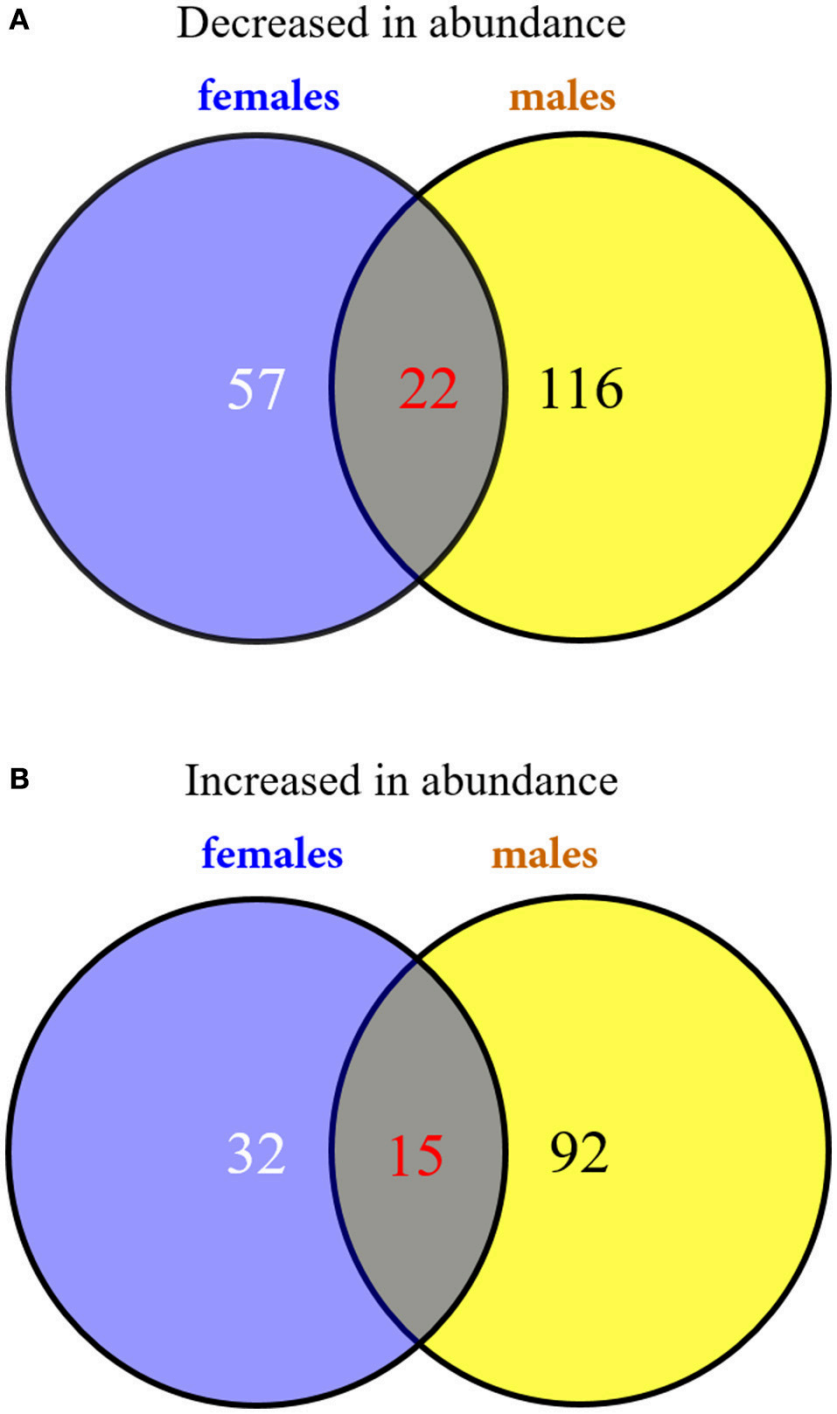
**Venn diagrams of the DEPs in ***P. cathayana*** males and females under high solar UV-B radiation. (A)** the down-regulated DEPs in abundance; **(B)** the up-regulated DEPs in abundance.

To investigate the changes in the protein profiles in response to solar UV-B radiation, proteins were extracted from the poplar leaves and analyzed using an iTRAQ-based shotgun proteomics strategy. Using the Mascot search engine, a total of 6,091 peptides from the trypsin-digested proteins were identified; of these, 5,210 peptides were unique. A total of 2,405 proteins were identified. The protein information is listed in Supplementary Table 1. According to the selection criteria of expression abundance ≥1.5 (or ≤ 0.67) and *P* ≤ 0.05, 331 proteins were defined as DEPs. In females, high UV-B radiation was associated with the decreased abundance of 79 proteins and the increased abundance of 47 proteins (Figure 3 and Supplementary Table 2). In males, high UV-B radiation was associated with the decreased abundance of 138 proteins and the increased abundance of 107 proteins (Figure 3 and Supplementary Table 3). According to the identified protein IDs, decreases and increases were observed in 22 and 15 proteins common in both sexes, respectively (Figure 3 and Table 4).

**Table 4:** **The common detected DEGs in both males and females caused by high UV-B radiation**.

**Protein name^a^**	**Protein accession^b^**	**Gene accession^c^**	**Females^d^**	**Males^d^**	**Score**	**Sequence coverage**	**Peptide^e^**
**AMINO ACID METABOLISM**							
5-methyltetrahydropteroyltriglutamate–homocysteine methyltransferase	B9MYM2	Potri.004G190900.1	−0.84	−1.36	1278	22	13/5
5-methyltetrahydropteroyltriglutamate–homocysteine methyltransferase	B9HQI3	Potri.009G152800.2	−0.66	−0.74	1494	28.6	16/8
Glycine cleavage system H protein, mitochondrial	A9PG07	Potri.003G089300.2	0.87	1.47	253	23.6	3/2
**CARBOHYDRATE AND ENERGY METABOLISM**							
Pyruvate dehydrogenase E1 component subunit alpha	A9PF50	Potri.002G179500.1	−0.61	−0.89	96	4.8	2/2
Geranylgeranyl diphosphate reductase, chloroplastic	A9PH25	Potri.012G068800.1	−0.91	−0.57	384	17	6/6
ADP, ATP carrier protein, mitochondrial	B9GFF3	Potri.009G062200.2	−0.62	−0.84	556	22.7	8/4
Glucan endo-1,3-beta-glucosidase, basic isoform	B9H9J8	Potri.006G048100.1	−0.59	−0.89	507	9.9	7/3
Sucrose synthase	B9MT39	Potri.006G136700.1	−0.60	1.94	291	5.7	4/4
**TRANSLATION, TRANSCRIPTION, AND POSTTRANSCRIPTIONAL MODIFICATION**							
40S ribosomal protein S15	A9PCZ0	Potri.002G043200.1	−0.69	−1.11	164	23.7	2/2
40S ribosomal protein S19-3	B9H0J0	Potri.004G118800.1	−0.85	−1.08	184	25.9	3/2
60S ribosomal protein L10	F6GU72	Potri.013G159600.1	−0.67	−2.39	268	9.1	2/2
Calreticulin	A9PEC9	Potri.013G009500.1	−0.58	−1.83	508	36.4	10/6
Chaperonin CPN60-2, mitochondrial	B9GMI8	Potri.001G054400.1	−0.95	−0.68	491	17.1	8/3
Probable LRR receptor-like serine/threonine-protein kinase At5g45780	B9HG85	Potri.007G001000.1	−0.89	−1.25	149	5.1	3/3
Cucumisin	B9HT44	Potri.010G196800.1	0.97	2.01	297	7.9	4/2
Thioredoxin O1, mitochondrial	B9MUN0	Potri.001G159000.1	0.84	0.79	104	22.2	4/4
Peptide methionine sulfoxide reductase B3, chloroplastic	B9GJ03	Potri.001G286500.1	0.92	0.94	97	15	3/3
Stromal 70 kDa heat shock-related protein, chloroplastic (Fragment)	B9N758	Potri.003G006300.1	0.62	1.00	4411	35.3	20/5
Thiol protease aleurain	A9P9P1	Potri.006G141700.7	0.83	0.80	235	9.2	3/3
Peptidyl-prolyl cis-trans isomerase FKBP19, chloroplastic	B9GMU6	Potri.001G068300.1	0.78	1.06	251	16.3	3/3
Ribosome-recycling factor, chloroplastic	B9GSN2	Potri.002G052400.1	1.20	0.89	792	28.1	8/8
Glycine-rich RNA-binding protein 2, mitochondrial	B9P731	Potri.001G319800.1	0.87	0.67	335	30.6	2/2
**PHOTOSYNTHESIS**							
Chlorophyll a-b binding protein CP24 10A, chloroplastic	A9PFP4	Potri.001G210000.1	−0.81	−0.67	214	15.6	2/2
Photosystem I reaction center subunit XI, chloroplastic	A9PGA1	Potri.014G175600.1	−1.37	−1.44	105	14.4	2/2
**STRESS RESPONSE**							
MLP-like protein 423	B9HVC5	Potri.010G096000.1	−0.74	−2.70	145	21.7	4/4
Endochitinase PR4	B9IQS9	Potri.019G094000.1	−0.63	−0.64	341	19.3	3/2
**OTHERS**							
Fasciclin-like arabinogalactan protein 8	B9IC18	Potri.014G071700.1	−0.58	−2.95	173	6.2	2/2
Fasciclin-like arabinogalactan protein 6	B9MW10	Potri.013G120600.1	0.71	−1.53	191	8.8	2/2
Omega-hydroxypalmitate O-feruloyl transferase	B9IFG0	Potri.015G100800.2	−0.65	−1.83	99	7.2	3/3
Histone H4	D7LK81	Potri.005G115600.1	−1.26	−1.38	128	24.4	4/4
ADP-ribosylation factor	F6HZD0	Potri.002G191400.1	−0.68	−0.66	448	22.7	5/5
Enoyl-[acyl-carrier-protein] reductase [NADH], chloroplastic	B9GLM6	Potri.001G013500.2	−0.77	−1.16	209	18.3	4/2
Phosphoribulokinase, chloroplastic (Fragments)	B9MUA4	Potri.001G134000.1	−0.66	−1.22	930	31.4	9/4
EG45-like domain containing protein	B9IM55	Potri.018G098200.1	1.77	1.30	144	39.2	3/3
Uncharacterized protein At4g13200, chloroplastic	A9PBY7	Potri.002G241000.1	0.91	1.34	381	34.9	4/4
Uncharacterized protein At2g27730, mitochondrial	B9MYW0	Potri.004G201700.1	1.06	1.43	109	20	2/2
14 kDa zinc-binding protein	B9IHG3	Potri.016G023700.1	0.79	1.37	292	26.9	3/3
Unknown protein DS12 from 2D-PAGE of leaf, chloroplastic	A9PHL9	Potri.008G010400.12	0.63	0.86	623	42	9/4
Unknown	A9PF14	Potri.013G007000.1	−0.83	−1.74	338	35.8	5/4
Unknown	A9PBQ7	Potri.005G076900.1	0.70	−0.94	141	24.6	4/3

a *The description in Uniprot_Swissprot database (http://www.uniprot.org/)*.

b *The accession number in Uniprot_Swissprot database*.

c *The accession number in poplar genome database (http://www.phytozome.net/Populus trichocarpa v3.0)*.

d *The changed folds of protein abundance (log_2_ transform)*.

e *The number of identified peptides and unique peptides*.

### Functional classification of DEPs

**Figure 4:**
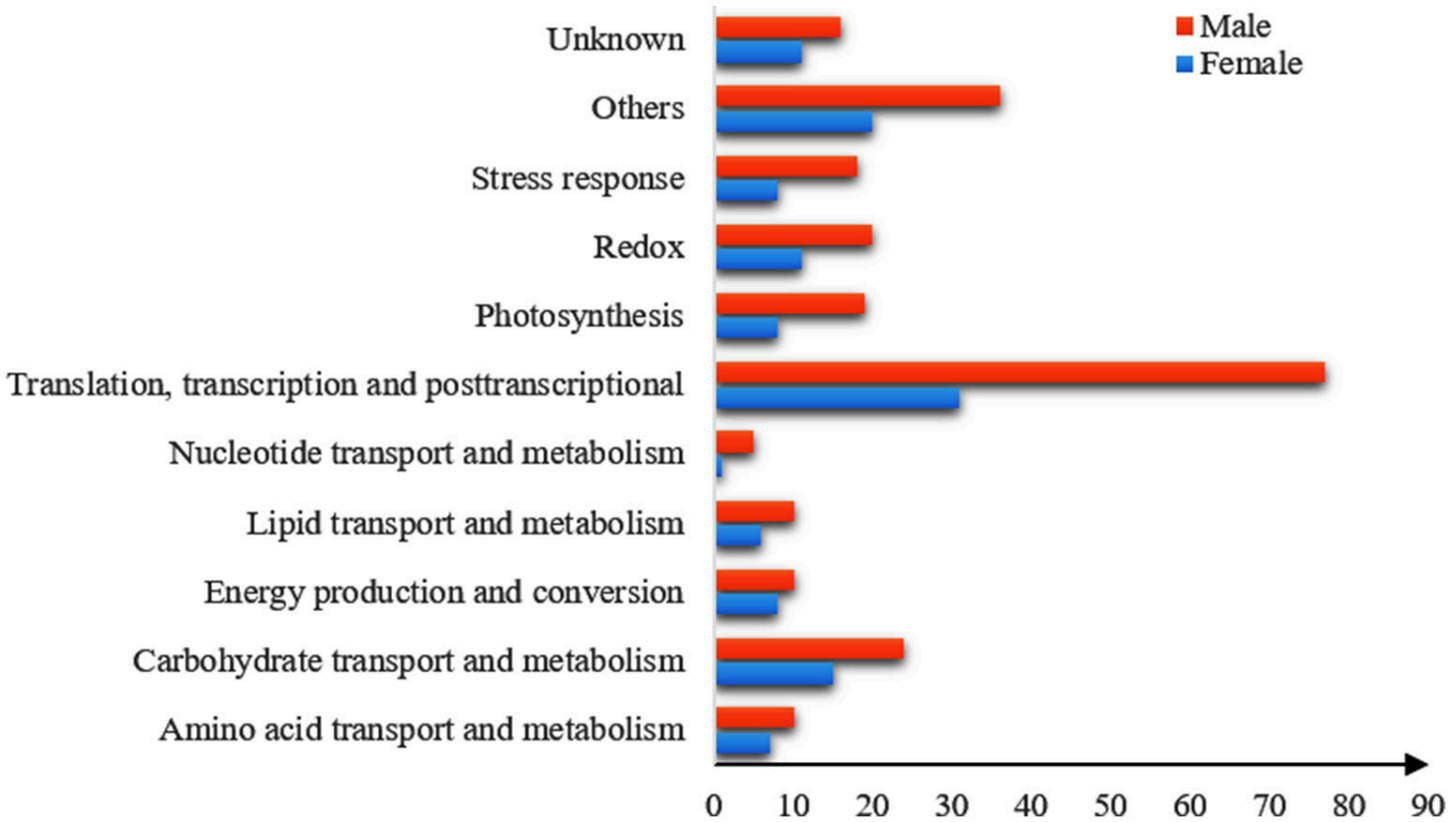
**Functional classification of the DEPs in males and females under the high solar UV-B radiation**. X-axis presents the number of DEPs. Y-axis presents functional classification of the DEPs.

**Figure 5:**
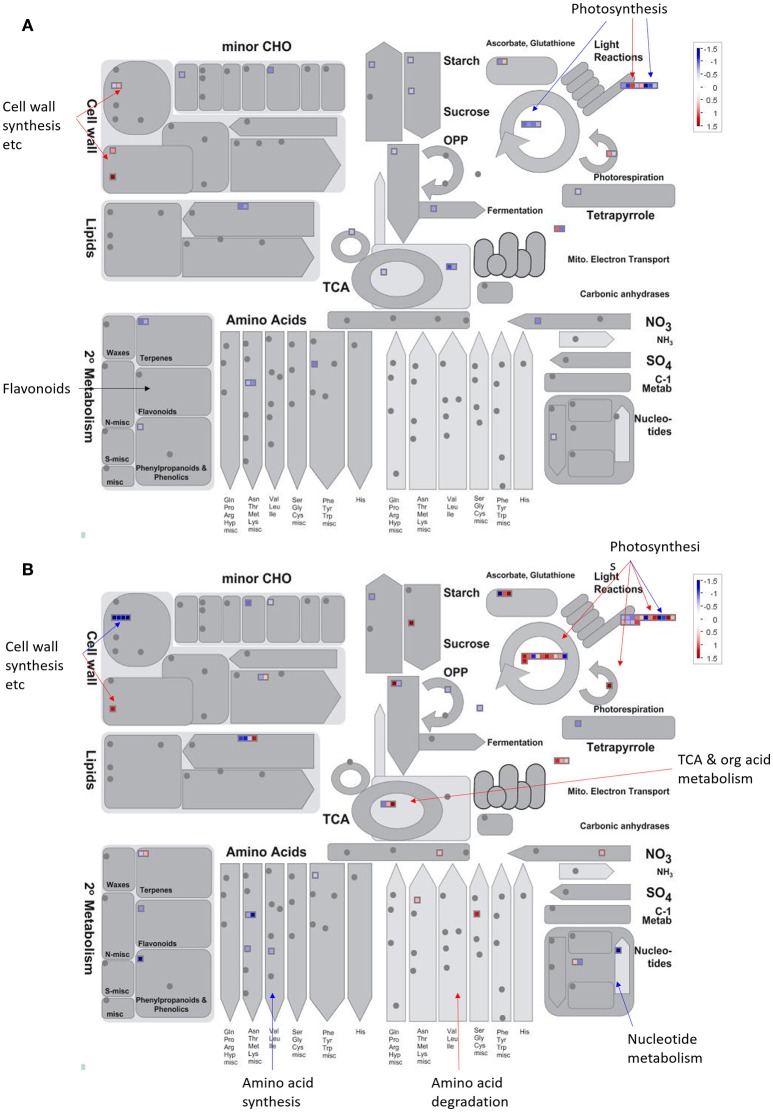
**An overview of the DEPs assigned to “metabolism” by MapMan**. Changes of proteomic level in **(A)** males and **(B)** females under solar UV-B radiation. Data were processed according to the standard protocol of MapMan software. Red indicates DEPs significantly up-regulated whereas blue indicates down-regulated in response to solar UV-B radiation (see color scale).

All of the DEPs were functionally categorized using Blast2go, a web-based bioinformatics tool that groups proteins based on their GO annotations. According to the molecular functions listed on the UniProt and Gene Ontology websites, the DEPs were classified into 11 functional categories in both sexes (Figure 4). Among these, the common DEPs in both sexes were involved in carbohydrate and energy metabolism, translation/transcription/post-transcriptional modification, photosynthesis, and redox reactions (Table 4, Supplementary Tables 2, 3 and Figure 5). The sex-specific DEPs were mainly involved in translation/transcription/post-transcriptional modification, amino acid metabolism, and stress responses (Supplementary Tables 2, 3). Additionally, a greater number of DEPs was observed in males than in females in all the categories, particularly in the translation/transcription/post-transcriptional modification proteins.

## Discussion

### Common changes between the sexes in the proteome in response to UV-B radiation

The commonly changed proteins in both sexes under high UV-B radiation were mainly involved in translation/transcription/post-transcriptional modification, carbohydrate and energy metabolism, photosynthesis, and redox reactions (Supplementary Tables 2, 3). Of these, RNA-binding proteins play roles in the transport, stability, and translation of mRNA (Sahi et al., 2007). Several RNA-binding proteins (e.g., glycine-rich RNA-binding protein and glycine-rich RNA-binding protein 2) were up-regulated by UV-B radiation. Glycine-rich RNA-binding protein (GRP) is involved in pre-mRNA splicing, nucleocytoplasmic mRNA transport, mRNA stability and decay, and translation (Dreyfuss et al., 1993; Simpson and Filipowicz, 1996). It plays an important role in the process of regulating gene expression, principally at the post-transcriptional level. Glycine-rich RNA-binding protein 2 may play a role in RNA transcription, which is likely because of its RNA chaperone activity during the stress adaptation process (Kim et al., 2007; Zhang et al., 2012). Numerous peptidyl-prolyl cis-trans isomerases were up-regulated in both sexes, particularly in males under UV-B radiation. The stromal 70 kDa heat shock-related proteins were also increased in abundance in both sexes. Heat shock proteins perform a chaperone function by stabilizing new proteins to ensure correct folding or by aiding the refolding of damaged proteins. The up-regulation of peptidyl-prolyl cis-trans isomerases and stromal 70 kDa heat shock-related proteins may be an adaption to avoiding protein misfolding and promoting the formation of disulfide bonds in nascent proteins under stressed conditions (Marshall and Keegstra, 1992; Schönbrunner and Schmid, 1992). Additionally, in conjunction with rRNA, ribosomal proteins compose the ribosomal subunits involved in the cellular process of translation. The evidence showed that UV-B radiation damaged ribosomes by crosslinking cytosolic and chloroplast ribosomal proteins to RNA. Ribosomal damage that accumulated during the initial stage of UV-B radiation correlated with a progressive decrease in new protein production; however, *de novo* synthesis of some ribosome proteins increases in the later stage of UV-B radiation (Casati and Walbot, 2004). In our study, a set of cytosolic and chloroplast ribosomal proteins (40S and 60S) was down-regulated in both sexes under UV-B radiation. This decrease may be attributable to ribosome rearrangement. Thus, these changes may enhance the efficiency of protein translation under UV-B stress. Ribosome rearrangement has been reported in many stress studies and is thought to be essential in maintaining efficient translation under stress. In addition, the down-regulation of elongation factors (e.g., elongation factor 1-alpha, beta and Ts, and elongation factor 2) that participate in protein synthesis in the cell cycle and facilitate translational elongation in the ribosome suggests that protein synthesis was hindered under high UV-B radiation. Furthermore, UV-B radiation might change protein phosphorylation levels and accelerate the degradation of misfolded proteins as suggested by the observation that LRR receptor-like serine/threonine-protein kinase At5g45780 was down-regulated and several proteases (e.g., thiol protease aleurain and protease Do-like 8) were up-regulated. The former catalyzes protein phosphorylation at serine/threonine (Gao et al., 2010), and the latter plays a role in proteolysis and leads to the mobilization of nitrogen under stressed conditions. Therefore, the results indicated that UV-B radiation might have a complex effect on gene expression regulation in *P. cathayana*, and a large variety of changes in gene expression processes, particularly post-transcriptional regulation and post-translational modifications, occurs in poplars. However, further study is needed to test this hypothesis.

Under high UV-B radiation conditions, many biological processes, including ion transport, ROS scavenging, and UV-absorbing compound synthesis, require extra energy. ATP is produced via light-driven photosynthetic reactions in the thylakoid membrane, and it is primarily utilized in carbon fixation reactions (Yin et al., 2010). In this study, ATP synthase subunits were down-regulated in females but up-regulated in males, indicating that more ATP might be produced in males than in females under UV-B radiation. However, several ATP carrier- and exchange-related proteins were decreased in abundance in both sexes, e.g., 3-isopropylmalate dehydrogenase, ATPase and ADP/ATP carrier proteins, suggesting that energy metabolism was hindered by high UV-B radiation in both sexes. However, this result was contrary to other reports that these proteins were up-regulated in plants suffering from short-term UV-B radiation, such as *Lonicera japonica* and *Arabidopsis* (Yin et al., 2010; Zhang et al., 2013). One reason might be that these changes in the proteins involved in energy metabolism varied with plant species, stress duration and development. In another study, ATP was produced by light-driven photosynthetic reactions and carbohydrate metabolism. However, in this study, several key enzymes involved in these pathways were down-regulated by UV-B radiation, e.g., PSI, PSII, cytochrome b6, glyceraldehyde-3-phosphate dehydrogenase and glucan endo-1,3-beta-glucosidase. Additionally, the high UV-B treatment lasted for a long time (one growth season), and excessive energy production will lead to accumulation of ROS and plant cell damage. Therefore, the decrease in ATP production may be an adaption to high UV-B conditions in nature, especially under higher intensities.

The photosynthetic system and subcellular organelles are vulnerable to UV-B damage. Previous studies have shown that high UV-B radiation lowers chlorophyll pigment content, damages the photosystem, and distorts chloroplast structure in *P. cathayana*, thereby leading to a decline in *P*_*n*_ (Ren et al., 2006, 2010; Xu et al., 2010; Feng et al., 2014). The decrease in chlorophyll content also has a very distinct effect on chlorophyll-binding proteins and protein complexes, thus further hindering light capture and delivery (Varsano et al., 2006; Zhang S. et al., 2016). In this study, the abundance of many photosynthetic proteins, especially light-dependent reaction proteins, was changed in both sexes (Figure 5). For example, many chlorophyll a/b binding proteins (CABs) and PSI and PSII reaction center proteins were lower in abundance under solar UV-B radiation, whereas some thylakoid lumen proteins and PsbP domain-containing proteins were increased. CABs are photosynthetic proteins that are typically down-regulated in plants in response to enhanced UV irradiation (Taylor et al., 1996; Zhang et al., 2013). These proteins are responsible for binding to chlorophyll pigment molecules in light-harvesting complexes and transferring excitation energy during photosynthesis (Bassi et al., 1990; Jespersen and Huang, 2015). In plants, the degradation of the photosystem is linked to the remodeling of light-harvesting antenna, e.g., chlorophyll a/b binding proteins (Moseley et al., 2002; Zhang S. et al., 2016). The lower expression of photosystem reaction center proteins implies that UV-B radiation causes electron transport discordance and redox homeostasis imbalance between PSI and PSII (Joshi et al., 2011). The cytochrome b6f complex, which mediates electron transport between PSI and PSII, is also a thylakoid-bound protein that is an essential component of the electron transport chain and serves as the final electron acceptor in the cyclical electron flow pathway of photosynthetic light reactions (Munekaga et al., 2004; Cramer et al., 2006; Zhang Y. et al., 2016). The PsbP and thylakoid lumen proteins were up-regulated in both sexes, indicating that solar UV-B radiation may result in instability of the photosystem (Jiang et al., 2015). PsbP proteins are essential for the regulation and stabilization of PSII (Ifuku et al., 2005). Recent reports have indicated that thylakoid lumen proteins play roles in regulating thylakoid biogenesis and the activity and turnover of photosynthetic protein complexes, especially the PSII and NAD(P)H dehydrogenase-like complexes. However, the function of the majority of luminal proteins in *Populus* remains unknown (Järvi et al., 2013; Zhang S. et al., 2016). Additionally, the abundance of OEE and Calvin cycle proteins was increased in males only, indicating sexual differences in the response of the photosynthetic system (Figure 5). OEE and Calvin cycle proteins participate in the light-dependent and light-independent reactions of photosynthesis. The up-regulation of these proteins might increase the photosynthetic capacity of males, which could partially explain the higher photosynthetic rate in males than in females under high solar UV-B radiation.

### Sex-specific changes in the proteome under UV-B radiation

Although a common set of proteins was changed in both sexes, there were many of sex-specific DEPs (Figure 5). For example, the 26S proteasome non-ATPase regulatory subunit 1 was up-regulated in females, whereas other proteasomes and their subunits were up-regulated in males (e.g., cysteine proteinase 2 and 3, proteasome subunit beta type-2-A and -6, and subtilisin-like proteases). Calnexin homolog 1 and luminal-binding protein 5, which control the apparatus of incorrectly folded proteins in the endoplasmic reticulum, were down-regulated in males only. The sex-specific changes in these proteins may be attributed to two reasons: (1) sex-specific gene expression and regulation may lead to sex-specific protein abundance and (2) limited proteomic detection technology (e.g., the number of identified peptides per proteins is still low) increases the difficulty of identifying all of the protein profiles in the two sexes, especially for the alkaline proteins and membrane proteins at low abundance.

A considerable number of proteomic profiles indicated that more proteins involved in amino acid metabolism were up-regulated to a greater degree in males than in females under high UV-B radiation (Figure 5). This result is consistent with that of the transcript profiles (Jiang et al., 2015). Alanine aminotransferase 2 and glycine dehydrogenase [decarboxylating] participate in the degradation of L-alanine and glycine, and isoaspartyl peptidase/L-asparaginase 1 are involved in aspartate production. The up-regulation of these proteins indicates that alanine, glycine, and aspartate catabolic processes changed more in males upon UV-B radiation. The changes in hydrolyzed amino acid compound observed in this study also confirmed this phenomenon. Additionally, several lyases, e.g., glycerate dehydrogenase and LL-diaminopimelate aminotransferase, were down-regulated in females, and 3-dehydroquinate synthase, diaminopimelate decarboxylase 2 and N-carbamoyl-L-amino acid hydrolase were down-regulated in males. These lyases catalyze the different chemical reactions that utilize and produce special amino acids. Glycerate dehydrogenase participates in glycine, serine, and threonine metabolism and glyoxylate and dicarboxylate metabolism, and their down-regulation only in females indicates that these metabolic processes are regulated more stringently in females than in males. Our results provide further evidence that the female strategy of gene regulation differs from that of males at the proteomic level.

For the females, except for 2 proteins that changed in common in both sexes, most of the stress responsive proteins were down-regulated. For example, 12-oxophytodienoate reductase 3 catalyzes the biosynthesis of jasmonic acid (JA), which is important as a gene regulator for development and defense (Schaller et al., 2000; Tani et al., 2008). 1-aminocyclopropane-1-carboxylate oxidase 3 (ACO3) is an essential gene during plant senescence and development and may play a role in ethylene synthesis (Hunter et al., 1999). The auto-inhibited plasma membrane P-type H+ ATPases were activated by 14-3-3 proteins (Jahn et al., 2002). Additionally, actin-7, phosphate carrier protein and protein canopy-1 were down-regulated in females, and several proteins were also down-regulated in males including allene oxide cyclase 3 (AOC3), probable linoleate 9S-lipoxygenase 5 (LOX1.5) and two protein aspartic proteases in guard cell 1 (ASPG1). AOC3 is involved in the production of 12-oxo-phytodienoic acid (OPDA), a pre-cursor of JA. ASPG1 play essential roles in restricting bacterial growth, plant defense and drought avoidance (Willmann et al., 2011; Yao et al., 2012). Plant lipoxygenases may be involved in a number of diverse aspects of plant physiology, including growth and development, pest resistance, senescence, and the wound response (Kolomiets et al., 2000). The down-regulation of these proteins indicates that higher intensity UV-B radiation may induce a sex-specific decrease in the defensive ability of poplars. Interestingly, several stress-related proteins were up-regulated only in males, such as heavy metal-associated isoprenylated plant protein 26 (HIPP26), heme-binding-like protein At3g10130, pathogenesis-related protein 1A (PRP1A), and stable protein 1 (SP1). PRP1A are plant disease-resistance proteins and play roles in plant defense. HIPP26 and At3g10130 are metal-binding proteins and play roles in metal detoxification (Barth et al., 2009; Vanhee et al., 2011). High UV-B radiation resulted in an increase in these stress-response proteins in *P. cathayana* males but not in females. Thus, these proteins might play roles in enhancing UV-B resistance to a greater extent in *P. cathayana* males than females.

Our previous parallel studies have shown that at the physiological level, solar UV-B radiation significantly decreases the photosynthetic capacity of *P. cathayana*, with a greater decrease in females than males (Xu et al., 2010; Feng et al., 2014). At the transcriptome level, solar UV-B radiation induced changes in the expression of a set of genes, and sexual differences were observed. For example, many differentially expressed genes involved in amino acid metabolism are up-regulated in *P. cathayana* males but down-regulated in females (Jiang et al., 2015). However, a full understanding of poplar regulatory mechanisms under UV-B solar radiation remains to be attained. Sex-related adaption to solar UV-B radiation in *P. cathayana* is such a sophisticated process that we cannot identify all of the regulating pathways that play dominant roles. For instance, flavonoids were the most frequently reported secondary metabolites responding to UV-B radiation in plants (Bassman, 2004; Schreiner et al., 2012). Our previous transcript study detected two flavonoid genes in *P. cathayana* males (Jiang et al., 2015). However, we did not identify any protein related to flavonoid metabolism at the proteomic level in this study (Figure 5). Numerous studies have indicated that the correlation between protein abundance and mRNA transcript levels is limited in woody plants (Lippert et al., 2009; Zhang et al., 2010; Dong et al., 2016). It is widely assumed that post-transcriptional regulation and post-translational modification play important roles in translational efficiency, which leads to discordance between mRNA expression and protein abundance. Therefore, to further understand the evolutionary and adaption mechanisms of poplars to UV-B radiation, differences in the translation, post-transcriptional modification, and metabolism responses should be investigated.

## Conclusions

Our results showed that *P. cathayana* females and males under high UV-B radiation exhibited common and sex-specific responses in terms of physiology and proteome dynamics. These common responsive proteins in both sexes were mainly categorized into carbohydrate and energy metabolism, translation/transcription/post-transcriptional modification, photosynthesis and redox reactions. The sexually different responsive proteins were involved in translation/transcription/post-transcriptional modification, amino acid metabolism, and stress responses. *P. cathayana* males showed a greater scope of protein changes than females under high UV-B radiation, suggesting that males are more sophisticated in their response to UV-B radiation compared with females. Although our study is limited by the low number of replicates (three replicates each treatment), and the results may vary depending on plant growth conditions (e.g., temperature, condensation and available light spectrum), it reveals some dynamic and sex-specific changes in poplars at the proteomic level in response to high UV-B radiation and provides new insights into the mechanism of the male-biased sex ratio at high altitude, thus complementing existing knowledge.

## Author contributions

YunxiangZ was responsible for the analysis of proteomic data and parts of manuscript writing. LF did the field work, and she was also responsible for obtaining proteomic data and analysis of physiological parameters. HJ and YuanbinZ did much of the field work and the measurements. SZ had the initial research idea, was responsible for parts of manuscript writing and acquired the funding for the project which was done in his laboratory.

## Funding

This work was supported by the Excellent Young Scientist Program of the National Natural Science Foundation of China (NO. 31322014) and the National Natural Science Foundation of China (No. 31170572 and 31300512).

### Conflict of interest statement

The authors declare that the research was conducted in the absence of any commercial or financial relationships that could be construed as a potential conflict of interest.

## Supplementary material

Supplementary Figure 1 **The experimental site and greenhouses used for UV-B filter (A)**, and the morphology of female plants under low UV-B radiation **(B)**.

Supplementary Table 1 **The detailed information of total identified proteins in ***P. cathayana*** males and females under UV-B radiation**.

Supplementary Table 2 **All the identified DEPs in ***P. cathayana*** females caused by UV-B radiation**.

Supplementary Table 3 **All the identified DEPs in ***P. cathayana*** males caused by UV-B radiation**.
