# Supplementary figures and images for: Different Proteome Profiles between Male and Female Populus cathayana Exposed to UV-B Radiation
Source: Front Plant Sci. 2017 Mar 7;8:320. doi: 10.3389/fpls.2017.00320 (PMC5339244; doi:10.3389/fpls.2017.00320)

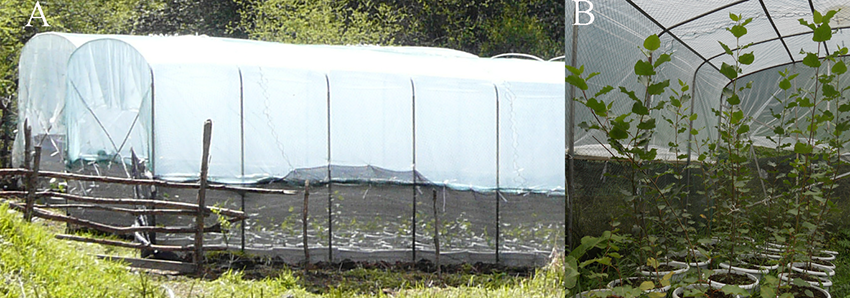

Supplement: Supplementary Figure 1 — The experimental site and greenhouses used for UV-B filter (A), and the morphology of female plants under low UV-B radiation (B). [file Image1.TIF]
